# Cost-effectiveness analysis of heart rate characteristics monitoring to improve survival for very low birth weight infants

**DOI:** 10.3389/frhs.2022.960945

**Published:** 2022-09-07

**Authors:** William E. King, Waldemar A. Carlo, T. Michael O'Shea, Robert L. Schelonka

**Affiliations:** ^1^Medical Predictive Science Corporation, Charlottesville, VA, United States; ^2^Department of Pediatrics, University of Alabama at Birmingham, Birmingham, AL, United States; ^3^Department of Pediatrics, University of North Carolina at Chapel Hill, Chapel Hill, NC, United States; ^4^Division of Neonatology, Department of Pediatrics, Oregon Health and Science University, Portland, OR, United States

**Keywords:** heart rate characteristics (HRC), cost-effectiveness analysis (CEA), very low birth weight (VLBW), neonatal intensive care unit (NICU), incremental cost-effectiveness ratio (ICER), randomized controlled trial (RCT), artificial intelligence (AI), newborn infant

## Abstract

**Introduction:**

Over 50,000 very low birth weight (VLBW) infants are born each year in the United States. Despite advances in care, these premature babies are subjected to long stays in a neonatal intensive care unit (NICU), and experience high rates of morbidity and mortality. In a large randomized controlled trial (RCT), heart rate characteristics (HRC) monitoring in addition to standard monitoring decreased all-cause mortality among VLBW infants by 22%. We sought to understand the cost-effectiveness of HRC monitoring to improve survival among VLBW infants.

**Methods:**

We performed a secondary analysis of cost-effectiveness of heart rate characteristics (HRC) monitoring to improve survival from birth to NICU discharge, up to 120 days using data and outcomes from an RCT of 3,003 VLBW patients. We estimated each patient's cost from a third-party perspective in 2021 USD using the resource utilization data gathered during the RCT (NCT00307333) during their initial stay in the NICU and applied to specific per diem rates. We computed the incremental cost-effectiveness ratio and used non-parametric boot-strapping to evaluate uncertainty.

**Results:**

The incremental cost-effectiveness ratio of HRC-monitoring was $34,720 per life saved. The 95th percentile of cost to save one additional life through HRC-monitoring was $449,291.

**Conclusion:**

HRC-monitoring appears cost-effective for increasing survival among VLBW infants.

## Introduction

The over 50,000 infants born each year in the United States with very low birth weight (VLBW; <1,500 g) are at high risk of mortality and morbidity and spend long periods in a neonatal intensive care unit (NICU) before discharge. Unsurprisingly, expenses to care for these premature patients are high, and the annual cost to care for these VLBW patients may exceed $10B in the US alone (1–7).

In one of the largest randomized controlled trials (RCT) ever conducted among VLBW patients, heart rate characteristics (HRC) monitoring in addition to standard monitoring decreased all-cause mortality by 22% (HR = 0.78; absolute risk from 10.2 to 8.1%) when compared with patients randomized to standard monitoring alone (8). Further, HRC-display was associated with a 40% reduction in mortality after infection (from 19.6 to 11.8%) (9) and improvement in survival without neurodevelopmental impairment among patients who developed a bacterial infection (10).

We sought to contextualize the costs of HRC-monitoring by undertaking a cost-effectiveness analysis of HRC-monitoring to improve survival using resource utilization and outcomes data from the RCT. The results may inform decision-making among clinicians caring for premature infants, hospitals undertaking technology assessments, third-party payers making coverage decisions, and nationalized health systems considering adoption of HRC monitoring.

## Methods

### Randomized controlled trial overview

As HRC-monitoring has been used as an indicator of the inflammatory status of NICU patients and as an early warning of infection (11–14), VLBW patients at eight study centers (nine hospitals) were randomized to either receive standard of care monitoring or standard of care monitoring *plus* HRC-monitoring (HeRO monitors provided by Medical Predictive Science Corporation, Charlottesville, Virginia, USA) using blocked allocation at each site using computer-generated sequences (NCT00307333). The trial was pragmatic in design; that is, there were no mandated interventions based on HRC. Rather, patients were randomized to receive either HRC-display or non-display while in the NICU during the subsequent 120 days, and then outcomes were tracked, including duration of mechanical ventilation, length of stay, antibiotic usage, and mortality. In the prespecified analyses, patients in the HRC-display group experienced reduced mortality in the 120-days after randomization when compared with controls (10.2 vs. 8.1%, *p* = 0.04) (8). This RCT had mortality data on 99.5% of the randomized patients in addition to resource utilization data including NICU length of stay and daily ventilatory status over a 120-day time horizon and post-discharge follow-up where appropriate, providing the opportunity to evaluate cost-effectiveness of the intervention.

### Analysis of cost-effectiveness

We performed a retrospective analysis of cost-effectiveness of HRC-monitoring to improve survival from a third-party payer's perspective where we included the direct medical costs and overhead from birth to NICU discharge, up to 120 days since birth, for patients in the RCT. This time horizon is typical of NICHD Neonatal Research Network studies, and includes ~80% of the costs of care and 98–99% of infant mortality in the premature infant population (15–19).

We estimated each patient's cost adjusted to 2021 USD using the resource utilization data from the case report forms gathered daily during the NICU stay and applied to per diem rates that include both hospital and physician services for the level of acuity (20) used in previous economic evaluations of RCTs among premature infants (21, 22). We assigned a daily cost of care for each patient based on the postnatal age and level of acuity (mechanically ventilated vs. non-mechanically ventilated) as follows, Day 1: $4,241.00 or $1,442.44; Days 2–28: $3,562.42 or $1,163.87; Days 29+: $3,577.12 or $1,163.87. These costs were derived from a cost-effectiveness analysis of corticosteroid therapy (20), although we used only two levels of acuity (mechanically ventilated vs. non-mechanically ventilated), the cited study used three levels of acuity (mechanically ventilated, continuous positive air pressure, and no respiratory support). The per-diem hospital costs were derived from patient charge data including personnel charges, non-personnel charges, and allocated hospital overhead values, and were converted to cost using center-specific federal cost-to-charge ratios (21).

For patients randomized to HRC-display, we used information from the manufacturer to calculate costs associated with HRC-monitoring (the system can be leased from the manufacturer for a total cost of 2021 USD 3.25 per patient per day in the NICU with no additional equipment costs; in-person staff training costs are additional, but on-line staff training is provided at no cost. For the purposes of this analysis, only the $3.25/day cost is used). This cost was not attributed to patients in the non-display group.

We computed the incremental cost-effectiveness ratio by calculating the difference in mean cost between the patients randomized to standard monitoring *plus* HRC-display vs. standard monitoring alone and dividing by the difference in survival between the two study groups. Hence, the incremental cost-effectiveness ratio can be interpreted as the total direct third-party costs in the first 120 days to save one additional life of a very low birth weight infant through HRC-monitoring.

We used non-parametric bootstrapping to evaluate the uncertainty of these estimates. In each of 5,000 simulations, we sampled each patient with replacement. For each simulation, we calculated the difference in mean survival between the two arms of the RCT, the difference in the mean cost, the incremental cost-effectiveness ratio, and the cost per survivor for the HRC-display and non-display patients. We report the results of the bootstrapping analysis as the 95th percentile, corresponding to a traditional confidence interval upper limit.

In addition, we performed deterministic sensitivity analyses by varying (a) the cost of HRC-monitoring, (b) the per-diem cost of time in the NICU, each by 25, 50, 200, and 400%, and then recomputing the incremental cost-effectiveness ratio, (c) varying the ratio of ventilated costs by 25, 50, 200, and 400% while holding non-ventilated costs constant, and (d) varying the ratio of non-ventilated costs by 25, 50, 200, and 400% while holding ventilated costs constant. The latter two address possible changes in ventilatory practice patterns since this dataset was generated. Finally, we corrected for changes in care since the completion of the RCT by decreasing mortality by 20% (23, 24) and recalculating the ICER.

To provide additional context, we evaluated the cost-effectiveness of HRC-monitoring from a *hospital* perspective, where we converted reimbursable costs to expected charges using the mean cost-to-charge ratio for all US hospitals.

All cost and charge data are presented in 2021 US Dollars. Conversions to US dollars were performed using exchange rates at the time of conversion as published by the US Treasury (for those studies in the Discussion that are used as comparisons) (25), and adjustments to account for inflation were performed using the US Bureau of Labor Statistics Medical CPI (Medical care in U.S. city average, all urban consumers, not seasonally adjusted) (26). All statistical calculations were performed in R (27). We followed the Consolidated Health Economic Evaluation Reporting Standards (CHEERS) (28).

## Results

Table 1 shows baseline demographics and outcomes for the patients randomized to standard of care monitoring (non-display) and standard of care monitoring *plus* HRC-monitoring (HRC-display). The report of the RCT found no statistically significant differences in baseline characteristics between the two arms of the study (8).

**Table 1:** Demographics, mortality, and resource utilization by randomization arm.

**Variable**	**Statistics**	**Non-display (*n* = 1,489)**	**HRC-display (*n* = 1,500)**	***P-*value**
Birth weight (grams)	Mean (SD)	986 (290)	999 (283)	0.220
Gestational age (weeks)	Median (Q1–Q3)	28 (26–30)	28 (26–30)	0.545
Male	Count (percent)	767 (51.5)	791 (52.7)	0.527
1-Min Apgar	Median (Q1–Q3)	5 (3–7)	5 (3–7)	0.567
5-Min Apgar	Median (Q1–Q3)	8 (6–8)	8 (6–8)	0.790
Survived	Count (Percent)	1,332 (89.5%)	1,375 (91.7%)	0.045
NICU length of stay (days)	Median (Q1–Q3)	59 (37–90)	60 (38–90)	0.458
Ventilated on day 1	Count (Percent)	617 (41.4%)	610 (40.7%)	0.696
Ventilated days 2–28 (days)	Median (Q1–Q3)	1 (0–12)	1 (0–13)	0.962
Ventilated days 29+ (days)	Median (Q1–Q3)	0 (0–3)	0 (0–3)	0.845
Total cost ($)	Mean (SD)	$108,066 ($86,877)	$108,834 ($85,454)	0.808
Cost of HRC ($)	Mean (SD)	$0 ($0)	$208 ($108)	N/A
Cost of NICU ($)	Mean (SD)	$108,066 ($86,877)	$108,626 ($85,362)	0.859
Cost of ventilated days ($)	Mean (SD)	$50,571 ($84,798)	$50,068 ($82,454)	0.869
Cost of non-ventilated days ($)	Mean (SD)	$57,495 ($31,845)	$58,558 ($30,401)	0.351

A 2-sided t-test is used to assess the significance of continuous variables, a Wilcoxon rank-sum test for ordinal variables, and a test for equality of proportions for categorical variables.

Total cost of care for patients randomized to HRC-display was $108,834 (SD: $85,454) vs. $108,066 (SD: $86,877) for non-display. HRC-monitoring alone added a mean cost to HRC-display patients of $208 (SD: $108). There was a longer median length of stay among HRC-display patient [60 days (IQR: 38–90) vs. 59 days (IQR: 37–90)].

Using a time horizon of 120 days, this analysis of cost-effectiveness revealed small increases in both mean cost ($768/patient) and effectiveness (2.2% absolute increase in survival) for patients randomized to HRC-display. From these figures, we calculated the incremental cost-effectiveness ratio as $34,720 per life saved.

**Figure 1:**
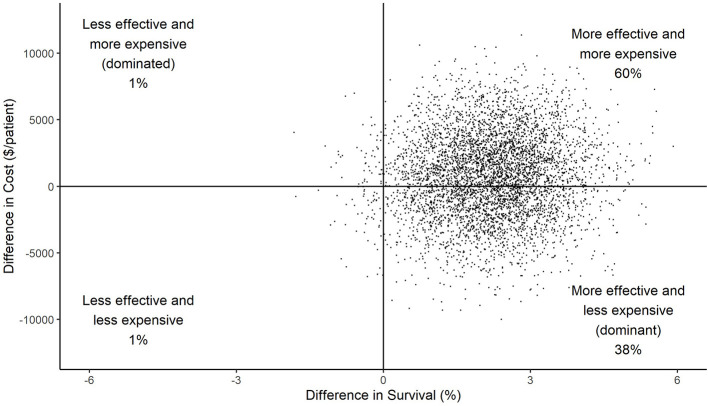
Scatterplot of difference in costs vs. difference in effect (survival) between HRC-display and non-display arms for each of 5,000 simulations. The large majority of points in the right half of the graph indicates a high level of confidence in the conclusion that HRC-monitoring was effective at reducing mortality; the smaller majority of data in the top half indicates a lower level of confidence that HRC-monitoring resulted in increased costs.

Figure 1 shows the results of 5,000 bootstrapped replications of the RCT with difference in cost plotted against difference in effectiveness (survival) for each simulation. HRC-monitoring was effective in improving survival in 98% of the replications, and less expensive in 39% of the replications. In 38% of the replications, HRC-monitoring was both more effective and less expensive (dominant). Whereas, in 1% of the replications, HRC-monitoring was less effective and more expensive (dominated). The highest proportion of replications, 60%, indicated HRC-monitoring to be both more effective and more expensive.

**Figure 2:**
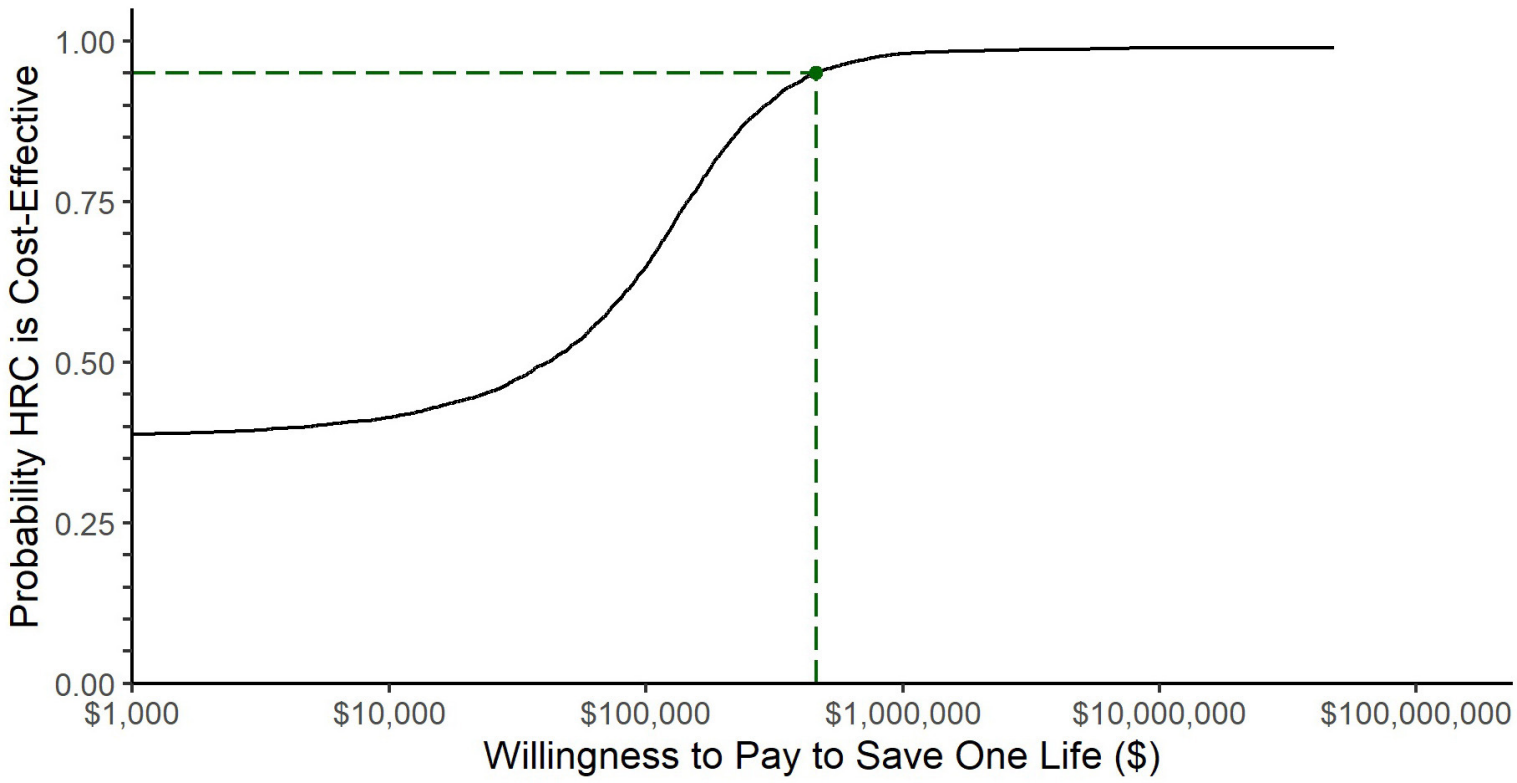
Cost-effectiveness acceptability curve. The black line represents the fraction of bootstrap replications for which HRC-monitoring was cost effective at the willingness-to-pay threshold represented along the x-axis. The dashed line represents the willingness-to-pay threshold at which 95% of simulations returned a lower incremental cost-effectiveness ratio: $449,291.

Figure 2 plots the cost effectiveness acceptability curve which shows the probability that HRC-monitoring is cost-effective at a range of thresholds of willingness-to-pay to save one life. The 95th percentile, corresponding to a traditional confidence interval limit, was $449,291, plotted with a dashed line.

**Figure 3:**
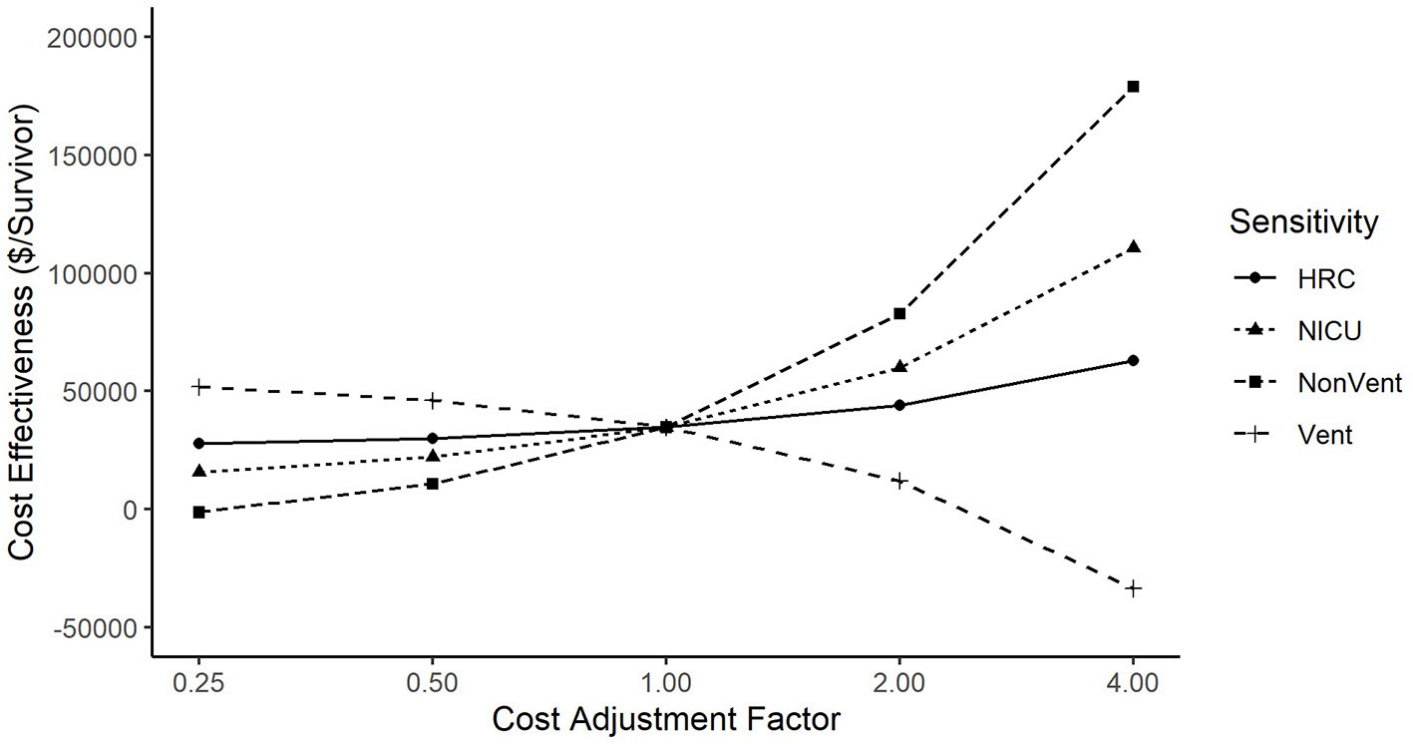
Sensitivity of incremental cost effectiveness ratio to variation in costs. Cost-effectiveness was more sensitive to changes in NICU Costs than to changes in HRC Costs. When analyzing ventilated vs. non-ventilated as separate components of NICU cost, cost-effectiveness varied directly with ventilated costs, but inversely with non-ventilated costs (that is, cost per life saved went down when ventilated costs went up, and cost per life saved went up when non-ventilated costs went up).

The incremental cost-effectiveness ratio was sensitive to changes when we varied both HRC cost (from 0.25x to 4x) and NICU cost (also from 0.25x to 4x). Figure 3 shows that the incremental cost-effectiveness ratio ranged from $27,668 per life saved when HRC costs were adjusted by 0.25x, to $62,927 when HRC costs were adjusted by 4x. The incremental cost-effectiveness ratio ranged from $15,732 when NICU costs were adjusted by 0.25x, to $110,674 when NICU costs were adjusted by 4x. Furthermore, when we broke NICU costs down into ventilated costs and non-ventilated costs, we saw that varying non-ventilated costs caused cost-effectiveness to range from –$1,341 per life saved (where a negative value indicates that the intervention both reduces costs and improves survival) at 0.25x to $178,966 per life saved at 4x, while holding ventilated costs constant. When holding non-ventilated costs constant, cost-effectiveness ranged from $46,102 per life saved when ventilated costs were adjusted by 0.25x to –$33,572 when ventilated costs were varied by 4x. The difference in sign between ventilated and non-ventilated days is explained by non-significant trends toward more ventilated days in the non-display patients, yet more non-ventilated days in the HRC-display patients. We note that the highest figure in the sensitivity analyses, $178,966 per life saved, was obtained when non-ventilatory days were raised by 4x, an adjustment that would make non-ventilated days higher in cost than ventilated days, an implausible scenario. Finally, when we decreased baseline mortality by 20% to adjust for trends in survival since the completion of the RCT, we found the incremental cost-effectiveness ratio increased to $43,400 per life saved.

**Figure 4:**
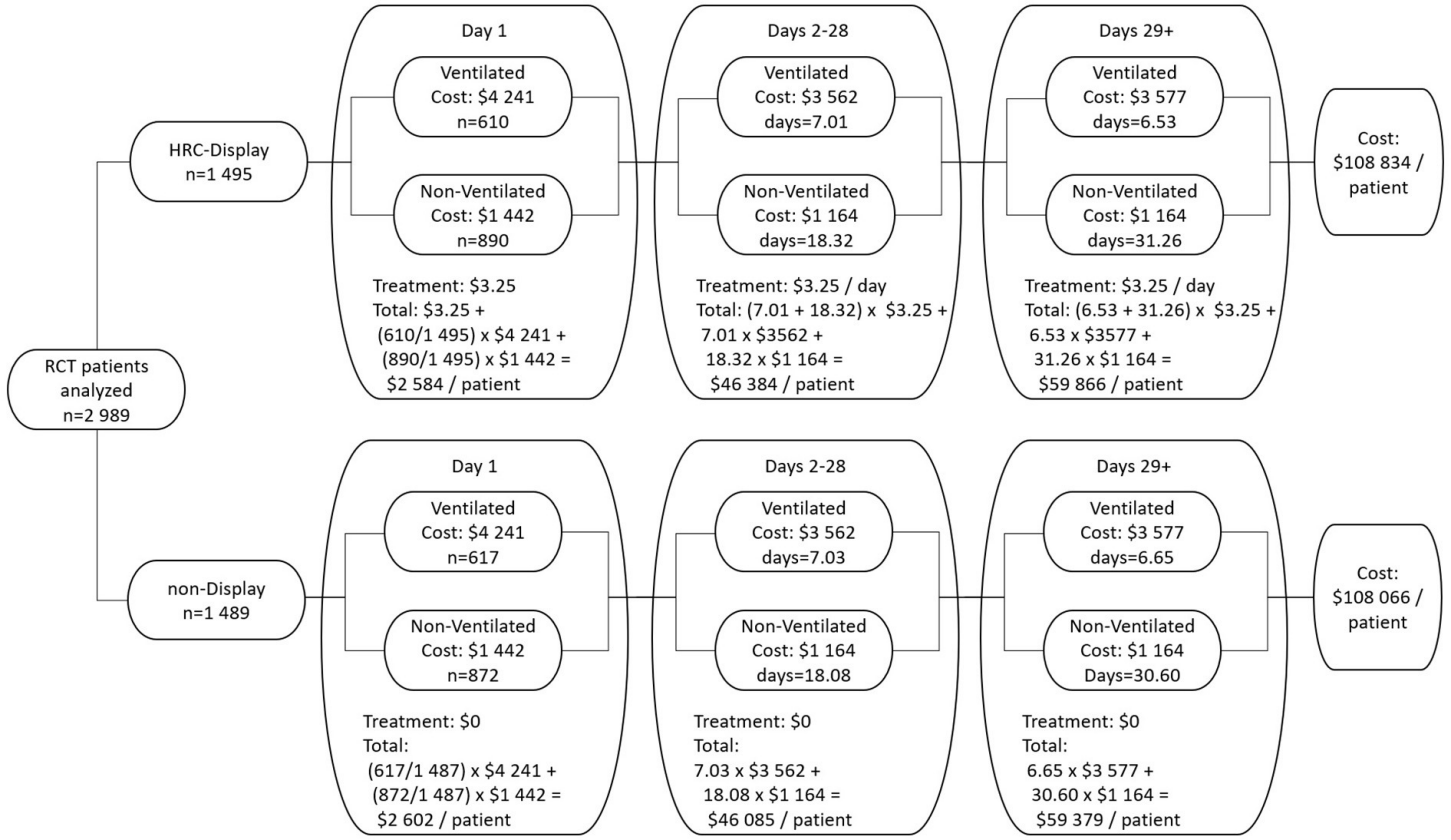
Cost decision tree.

From a hospital perspective, the difference in chargeable costs per patient is (1 – 0.08) × ($108,626–$108,066) (Table 1), or $515 per patient, after assuming that 8% of total per diem costs are represented by physician services, which are not reimbursable to the hospital (22). Using the median cost-to-charge ratio of US hospitals of 0.25 (29), a hospital could expect to be reimbursed for $515/0.25, or $2,061. Hence, the net profit from a hospital perspective to HRC-monitor one VLBW patient is $2,061–$515–$208, or $1,338 per VLBW patient. Figure 4 details the cost decision tree.

## Discussion

Using data from a large RCT conducted among VLBW infants, we estimated the third-party cost per life saved with HRC-monitoring, $34,720. This figure is roughly equivalent to the total cost of caring for infants born at 34-weeks gestational age as reported in a study of birth hospitalization costs in California, and more cost-effective per life saved than caring for a baby of any gestational age category below 34 weeks. Notably, in the same report, the cost per survivor (mean cost per patient divided by rate of survival) for the 23- and 24-week gestational age averaged $700,000–$750,000 in 2021 dollars (7).

The primary driver of the incremental cost to save a life through HRC-monitoring was not the $208 mean cost per patient of HRC-monitoring, which comprised roughly one quarter of the $768 mean cost difference. Instead, the primary cost driver of saving a life through HRC-monitoring was the additional time spent in the NICU, because more patients survived to discharge in the HRC-display arm.

Clinicians evaluating the cost-effectiveness of NICU HRC monitoring may apply these data to their unit from a *hospital perspective*. HRC-monitoring can be leased from the manufacturer at $3.25 per day per occupied bed. For a 20-bed unit with 80% occupancy the estimated annual cost to HRC-monitor all beds is $3.25 × 20 × 80% × 365, or $18,980 per year. The number needed to treat to save one VLBW life through HRC-monitoring is 48 (8). Assuming that only VLBW patients receive a benefit from HRC-monitoring even though all NICU patients would be monitored, if a 20-bed unit admits 48 VLBW patients per year, then one additional life would be saved at the cost of $18,980 for HRC-monitoring plus chargeable costs of $24,730, that is 48 × (1 – 0.08) × ($108,626 – $108,066), assuming that physician services comprise 8% of per diem costs (22). If the hospital's cost-to-charge ratio is a typical 0.25 (27), the hospital could be expected to bill third-party payers for an additional $2,061 per VLBW patient, that is (1 – 0.08) × ($108,626 – $108,066)/0.25, or $98,918 annually. Hence, the net profit to the hypothetical 20-bed NICU to implement HRC-monitoring is $55,209 per year, that is $98,918–$18,980–$24,730, and would result in one additional life saved per year. Universal HRC-monitoring of NICU patients is likely to *improve* the top-line and bottom-line of the hospital while survival also improves.

Parental costs either during or after NICU stay were not included, and thus we were unable to evaluate cost-effectiveness from a societal perspective. Other studies indicate that lost time at work is the primary driver of parental costs (22), which would be likely to have been highly correlated with length of stay in the NICU during the 120-day time horizon of this analysis, just as the other drivers of cost that were included in this analysis (HRC-monitoring is priced per day in the NICU, and our cost model of resource utilization is also primarily driven by length of NICU stay). Hence, results when evaluating from a societal perspective are unlikely to be substantively different than those from a third-party payer's perspective.

Some new therapies offer both reduced costs *and* improved outcomes. Antenatal betamethasone was found to decrease both costs of care and respiratory morbidity (20), and caffeine treatment for apnea of prematurity was found to both lower costs and decrease death or neurodevelopmental impairment (30).

More often, new interventions are expensive when they are introduced, and the *incremental* cost to improve an outcome is much higher than the *average* cost of care for the patients without the new technology. Inhaled nitric oxide is labeled for use among term infants with persistent pulmonary hypertension where it was found to improve survival at a cost of $56,132 per survivor (31). While the American Academy of Pediatrics recommended against the routine use of inhaled nitric oxide for premature infants (32), the NO CLD study did find that inhaled nitric oxide was cost-effective at reducing the composite outcome of death or bronchopulmonary dysplasia among premature infants at an estimated cost of $29,696 to reduce one case of death or bronchopulmonary dysplasia (21). Extracorporeal membrane oxygenation among neonates with severe respiratory failure was found to cost $38,885 per *year of life* saved (converted from pound sterling) (33), universal hearing screening was found to cost $80,691 per case of deafness (34), and donor human milk was found to cost $4,766 per averted case of necrotizing enterocolitis (converted from Canadian dollars) (15). In light of these commonly used neonatal interventions, universal HRC-monitoring among VLBW patients would appear to be a cost-effective means of improving an important outcome.

Strengths of this analysis include a large dataset collected under auspices of an RCT protocol at multiple centers throughout the US including outcomes data along with utilization data. Weaknesses of this analysis include the inability to estimate costs from a societal perspective, and the retrospective analysis of a dataset collected when rates of infection and ventilatory practices were different, and which have likely influenced the incidence, presentation, and response to sepsis in positive and negative ways. Further, the indirect method of estimating patients' costs through utilization records based primarily on ventilatory status rather than directly collecting cost data could have masked differences in resource utilization and cost, more so than the referenced studies (20–22) that used three levels of respiratory support as opposed to two. While the relatively short time-horizon of 120 days adequately captures changes in length of stay in the NICU associated with HRC-monitoring in this population where median length of stay was ~60 days, it may fail to capture life-long costs associated with survival after prematurity and/or increased survival associated with HRC-monitoring (35) (we note that we have found no difference in the rate of neurodevelopmental impairment among ELBW survivors of sepsis that were HRC-monitored compared with those that were not (29 vs. 29%, respectively, *p* = 0.95) (10), or among all ELBW survivors that were HRC-monitored compared to those that were not (19.8 vs. 17.9%, respectively, *p* = 0.63) (35), offering some expectation that future costs related to the increased survivorship after HRC-monitoring are not higher than the costs of other NICU survivors). Finally, the RCT was conducted in the United States with distinct patient populations, standards of care, and reimbursement models that could limit generalizability. Since the RCT, HRC-monitoring has been adopted in NICUs worldwide, and a recent study from the Netherlands indicates HRC-monitoring improved morbidity associated with sepsis (decreased nSOFA, *p* = 0.01) with a trend toward improved survival (*p* = 0.13) (36). This provides some evidence that the RCT results are generalizable to new patient populations and to current standards of care. Further, the trend that primarily drove the cost difference in our analysis, that is increased survival resulting in 1-day longer median length of stay, seems likely to also result in cost-effectiveness in regions with systems of socialized medicine.

Our estimate of the *incremental* cost to save a life with HRC-monitoring is lower than the *average* cost of caring for patients in the NICU without HRC-monitoring. It follows that adopting HRC-monitoring may actually improve the cost-effectiveness of NICUs to save lives. The cost-effectiveness of HRC-monitoring for increasing survival among very low birth weight infants is comparable to, or lower than, other interventions used routinely in the neonatal intensive care unit.

## Data availability statement

The original contributions presented in the study are included in the article/supplementary material, further inquiries can be directed to the corresponding author.

## Author contributions

WK was primarily responsible for the conception and first draft of the work. RS, WC, and TO'S were responsible for the acquisition of the data and interpretation of the work. All authors worked together on revising the work critically for important intellectual content, gave final approval of the version to be published, and agree to be accountable for all aspects of the work.

## Funding

The original randomized controlled trial was supported by the NIH Grant R01-HD48562 to WC, TO'S, and RS and by MPSC (WK). Open access publication fees were provided by MPSC.

## Conflict of interest

Author WK was employed by and has equity shares in Medical Predictive Science Corporation, Charlottesville, VA. The remaining authors declare that the research was conducted in the absence of any commercial or financial relationships that could be construed as a potential conflict of interest.

## Publisher's note

All claims expressed in this article are solely those of the authors and do not necessarily represent those of their affiliated organizations, or those of the publisher, the editors and the reviewers. Any product that may be evaluated in this article, or claim that may be made by its manufacturer, is not guaranteed or endorsed by the publisher.
